# Strong coupling among semiconductor quantum dots induced by a metal nanoparticle

**DOI:** 10.1186/1556-276X-7-95

**Published:** 2012-02-01

**Authors:** Yong He, Ka-Di Zhu

**Affiliations:** 1 Key Laboratory of Artificial Structures and Quantum Control (Ministry of Education), Department of Physics, Shanghai Jiao Tong University, 800 DongChuan Road, Shanghai 200240, China

## Abstract

Based on cavity quantum electrodynamics (QED), we investigate the light-matter interaction between surface plasmon polaritons (SPP) in a metal nanoparticle (MNP) and the excitons in semiconductor quantum dots (SQDs) in an SQD-MNP coupled system. We propose a quantum transformation method to strongly reveal the exciton energy shift and the modified decay rate of SQD as well as the coupling among SQDs. To obtain these parameters, a simple system composed of an SQD, an MNP, and a weak signal light is designed. Furthermore, we consider a model to demonstrate the coupling of two SQDs mediated by SPP field under two cases. It is shown that two SQDs can be entangled in the presence of MNP. A high concurrence can be achieved, which is the best evidence that the coupling among SQDs induced by SPP field in MNP. This scheme may have the potential applications in all-optical plasmon-enhanced nanoscale devices.

## 1 Introduction

Due to the advances in modern nanoscience, various nanostructures such as metal nanopartities (MNPs), semiconductor quantum dots (SQDs) and nanowires can be constructed for the applications in photonics and optoelectronics [1,2]. Studies of these nanostructures are essential for further development of nanotechnology. MNPs can be excited to produce surface plasmon polaritons (SPP) [3]. The energy transfer effect in a hybrid nanostruction complex composed of MNPs and SQDs has been observed, which implies the light-matter interaction between SPP field in MNPs and the excitons in SQDs [4,5]. To display the interaction between the exciton and SPP field, the vacuum Rabi splitting has been studied theoretically [6,7] and experimentally [8]. However, in the SQD-MNP coupled system a nonlinear Fano effect can be produced by a strong incident light [9]. Various theoretical [10,11] and experimental [12-14] reports have shown a decrease of the exciton lifetime of SQD placed in the vicinity of MNP. The decrease is related to the distance between SQD and MNP as a result of the coupling of the exciton and SPP field [15]. Moreover, the exciton energy level of SQD can be shifted because of the influence of SPP field [14]. Recently, the coupling among SQDs mediated by SPP field has received increasing attention [16,17]. The complex system like cavity QED system [18] and circuit QED system [19] may be applied in quantum information. Owing to the advantages of the solid-state of SQDs and integrated circuits of these nanostructures, the complex system is a promising candidate to implement the quantum information processing. However, more details about the coupling among SQDs and the role of SPP field need to be further studied. To illustrate clearly these quantum effects, a full quantum mechanics method to describe the coupled SQD-MNP system have to be developed.

In the present article, cavity QED as a quantum optics toolbox provides a full quantum mechanics description of the coupled SQD-MNP system. Under the description we develop a novel quantum transformation method that is suitable for the coupling SQDs to SPP field with large decay rate. The quantum transformation is used to treat master equation of the entire system. Under a certain condition, we obtain an effective Hamiltonian in SQDs' subsystem, and show a modified decay rate for each SQD. The effective Hamiltonian demonstrates an exciton energy shift and the coupling among SQDs. A cross-decay rate is induced by SPP field. It not only changes the decay rate of each SQD but also makes decay between every two SQDs. We analyze the exciton energy shift and the cross-decay rate of every SQD and the coupling among SQDs, and find that these parameters are related to the distance between SQD and MNP. An experimental scheme to obtain these parameters is proposed by the observation of the signal light absorption spectrum of SQD in a system consisted of an SQD and an MNP. Based on the achievement of thes parameters, we design a simple model that two identical SQDs interact with an Au MNP for demonstrating the coupling of two SQDs.

## 2 Theory

We consider multiple SQDs in the vicinity of an MNP. Each SQD consists of the electronic ground state |0〉 and the first excited state |*ex*〉. They interact with SPP field in the MNP. First, we need to quantize SPP field based on the cavity quantum electrodynamics (QED). Recently, a good deal of study had been devoted to quantize SPP field in the metal [20-24]. SPP field in the MNP can be considered as a multiple-modes field. After the second quantization of SPP field, the Hamiltonian can be written as $H_{SPP}=\underset{k}{\sum }\omega_{k}a_{k}^{+}a_{k}$[20,21], where *ω*_*k *_is the frequency of SPP mode $k,a_{k}^{+}\left(a_{k}\right)$ is the creation (annihilation) operation of SPP mode *k*. Next, we consider the interaction between each SQD and SPP modes. We assume that the coupling strength between each SQD and SPP field is identical for simplicity. The interaction Hamiltonian, under the rotating-wave approximation, can be written as $H_{int}=-\underset{i,k}{\sum }\left(g_{k}a_{k}\sigma_{+}^{i}+g_{k}^{*}a_{k}^{+}\sigma_{-}^{i}\right)$[22,25], where *g*_*k *_is the coupling strength between each SQD and SPP mode $k,\sigma_{+}^{i}=|{ex\rangle }_{i}\langle 0|(\sigma_{-}^{i}=|{0\rangle }_{i}\langle ex|)$ is the raising (lowering) operator of the *i*th SQD. Therefore, the Hamiltonian of the entire system can be written as (*ħ *= 1)

(1) $$H=\underset{i}{\sum }\omega_{ex}\sigma_{z}^{i}+\underset{k}{\sum }\omega_{k}a_{k}^{+}a_{k}-\underset{i,k}{\sum }\left(g_{k}a_{k}\sigma_{+}^{i}+g_{k}^{*}a_{k}^{+}\sigma_{-}^{i}\right),$$

where $\sigma_{z}^{i}=(1/2)\times {(|ex\rangle }_{i}{\langle ex|-|0\rangle }_{i}\langle 0|)$. The full quantum dynamics of the coupled nanosystem can be derived from the following master equation for the density operation

(2) $$\partial_{t\rho }=-i\left[H,\rho \right]+\varsigma_{SQD}+\varsigma_{SPP},$$

with the Liouvillian terms [26,27], $\varsigma_{SQD}=\left(\kappa /2\right)\times \underset{i}{\sum }\left(2\sigma_{-}^{i}\rho_{+}^{i}-\rho \sigma_{+}^{i}\sigma_{-}^{i}-\sigma_{+}^{i}\sigma_{-}^{i}\rho \right)$ describes the decay of each SQD to Markovian reservoirs, *κ *is the exciton radiative decay rate in SQDs, $\varsigma_{SPP}=\underset{i}{\sum }\left(\gamma_{k}/2\right)\times \left(2a_{k}\rho a_{k}^{+}-\rho a_{k}^{+}a_{k}-a_{k}^{+}a_{k}\rho \right)$ describes the relaxation of SPP mode *k *with decay rate *γ*_*k*_. Next, we take a time-independent unity transformation *e*^*is *^on the density operator, where $s=\underset{i,k}{\sum }\left(\pi_{k}a_{k}\sigma_{+}^{i}+\pi_{k}^{*}a_{k}^{+}\sigma_{-}^{i}\right),\pi_{k}=2_{gk}/\left(\gamma_{k}+2i\delta_{k}\right),\delta_{k}=\omega_{k}-\omega_{ex}$, so that $\tilde{\rho }=e^{is}\rho e^{-is}$,

(3) $$\partial_{t}\tilde{\rho }=-i\left[e^{is}He^{-is},\tilde{\rho }\right]+e^{is}\varsigma_{SQD}e^{-is}+e^{is}\varsigma_{SPP}e^{-is}.$$

If |*π*_*k*_| ≪ 1, the second-order term remains, and the higher-order terms can be ignored safely. To obtain the reduce density operation of the SQDs' subsystem, we take a trace over the SPP field of the both hands of Eq. (3) by using *Tr*_*SPP*_[.]. Here, we assume that the multi-mode plasmon field can be consider as a thermal reservoir and the reservoir variables are distributed in the uncorrelated thermal equilibrium mixture of states, $<a_{k}>=<a_{k}^{+}>=0,<a_{k}^{+}a_{l}>={\bar{n}}_{k}\partial_{kl}$, where the thermal average boson number ${\left({\bar{n}}_{k}\right)}^{-1}=exp\left[\left(\omega_{k}\right)/\left(k_{B}T\right)\right]-1$, *k*_*B *_is the Boltzmann constant, and *T *is the temperature. Therefore,

(4) TrSPP[∂tρ˜]=−i[{∑iωexσzi+∑kωkn¯k+∑i2Re[πk(πk*δk+i2gk*)](2n¯k+1)σzi +∑i<j2Re[πk(πk*δk+i2gk*)](σ+iσ−j+σ−iσ+j)},ρSQD]+ςSQD+∑i,jIM[πk(πk*δk+i2gk*)](2σ−iρSQDσ+j−σ+iσ−jρSQD−ρSQDσ+iσ−j),

where $\rho_{SQD}=Tr_{SPP}\left[\tilde{\rho }\right]$. After some algebraic calculation, the master equation of the reduce density operation of the SQDs' subsystem can be written as

(5) $$\partial_{t}\rho_{SQD}=-i[H_{eff},\rho_{SQD}]+{\varsigma^{\prime }}_{SQD}.$$

The effective Hamiltonian to reveal the exciton energy shift and the coupling among SQDs is given by

(6) $$H_{eff}=\sum_{i}(\omega_{ex}-\eta_{0})\sigma_{z}^{i}-\eta \sum_{i<j}\left(\sigma_{+}^{i}\sigma_{-}^{j}+\sigma_{-}^{i}\sigma_{+}^{j}\right),$$

where $\eta_{0}=\eta +\underset{k}{\sum }{8|g_{k}|}^{2}\delta_{k}{\bar{n}}_{k}/\left(\gamma_{k}^{2}+4\delta_{k}^{2}\right),{\bar{n}}_{k}=<a_{k}^{+}a_{k}>$, and $\eta =\underset{k}{\sum }4|g_{k}{|}^{2}\delta_{k}/\left(4\delta_{k}^{2}+\gamma_{k}^{2}\right)$ are the coupling strength among SQDs induced by quantized SPP modes. We can see that *η*_0 _represents the exciton energy shift as a result of the coupling SQD to all quantized SPP modes. In the bosonic bath composed of all SPP modes, according to the Bose-Einstein distribution function, ${\bar{n}}_{k}\ll 1$ at low temperature so that $\eta_{0}≅\eta$. The dissipation term is given by

(7) $$\varsigma_{SQD}^{\prime }=\left(\Gamma_{i,j}/2\right)\times \underset{i,j}{\sum }\left(2\sigma_{-}^{i}\rho_{SQD}\sigma_{+}^{j}-\sigma_{+}^{i}\sigma_{-}^{j}\rho_{SQD}-\rho_{SQD}\sigma_{+}^{i}\sigma_{-}^{j}\right),$$

Γ_*i,j *_= *κ *+ *2τ *if *i *= *j*, Γ_*ij *_= 2*τ *if *i *≠ *j*, where $\tau =\underset{k}{\sum }2{\left|g_{k}\right|}^{2}\gamma_{k}/\left(4\delta_{k}^{2}+\gamma_{k}^{2}\right)$. We note that a cross-decay rate 2*τ *between every two SQDs appears and the exciton lifetime decreases because of the presence of SPP field. The cross-decay rate represents the nonradiative decay rate that can be decomposed into different contributions for each SPP mode, i.e., $2\tau ≅\Gamma_{MNP}^{nr}$[22].

Our method to treat the Hamiltonian is similar with Schrieffer-Wolff transformation [28]. In cavity (circuit) QED system, when the decay rate of cavity mode is very small as compared to the detuning between the cavity mode frequency and the transition frequency of qubits so that it can be ignored safely, the effective Hamiltonian can be obtained by using Schrieffer-Wolff transformation [18,19]. Under the treatment of Schrieffer-Wolf transformation, one can obtain $\eta =\underset{k}{\sum }{\left|g_{k}\right|}^{2}/\delta_{k},\tau =0$. But it is well-known that the decay of SPP field is too large to be ignored in the coupled SQD-MNP system. Taking this fact fully into account, our method is suitable for revealing the exciton energy shift, the modify decay rate and the coupling strength among SQDs.

## 3 Coupling an SQD to an MNP

**Figure 1:**
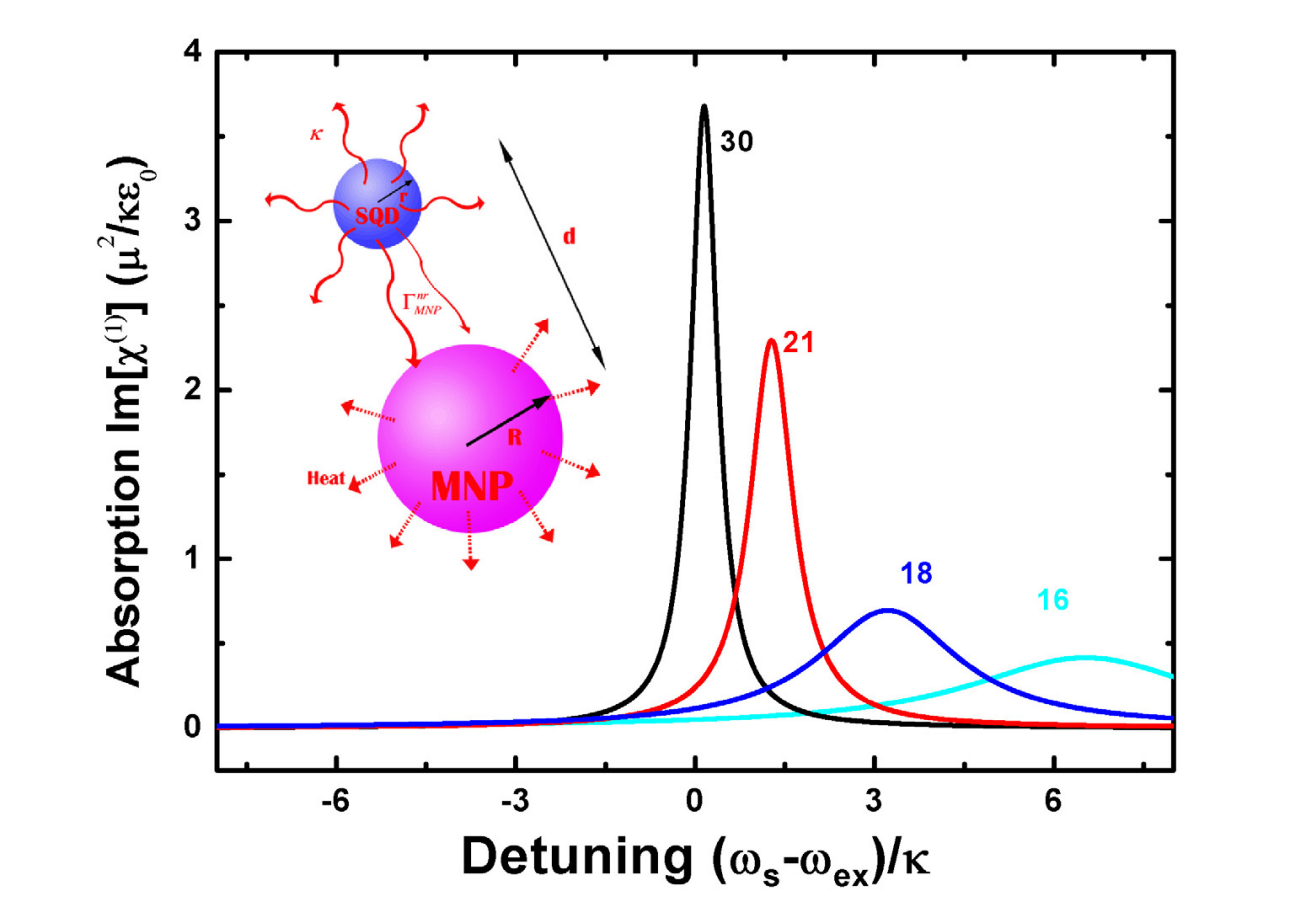
**The signal light absorption spectrum of SQD**. The signal light absorption spectrum of SQD for different distance *d*. The inset shows a complex system composed of a SQD to a MNP. A SQD with radius *r *is placed in the vicinity of a MNP with radius *R*. The center-to-center distance is *d*.

Now, we consider a simple complex system composed of an SQD and an MNP. As illustrated in inset of Figure 1, an SQD with radius *r *is placed in the vicinity of an MNP with radius *R*. The center-to-center distance is *d*. The modified decay rate of the SQD includes the radiative decay rate *κ *and the nonradiative decay rate $\Gamma_{MNP}^{nr}$ induced by MNP. Owing to the ohmic losses within the metal a significant fraction of absorbed power has be dissipated as heat [3]. We first estimate the parameters *η *and *τ*. In the complex system, the SQD can induce polarization of MNP $P_{MNP}=[\gamma s_{\alpha }R^{3}P_{SQD}]/[\varepsilon_{eff1}d^{3}]$, where $\gamma =[\varepsilon_{M}(\omega )-\varepsilon_{0}]/[\varepsilon_{M}(\omega )+2\varepsilon_{0}],\varepsilon_{eff1}=[\varepsilon_{s}+2\varepsilon_{0}]/[3\varepsilon_{0}],P_{SQD}=\mu (<\sigma_{+}>+<\sigma_{-}>)$[9], *ε*_0_, *ε*_*s*_, and *ε*_*M *_are the dielectric constants of the background medium, the SQD and the MNP, respectively, *μ *is the electric dipole moment of the exciton, *s*_*α *_is related to the direction of the coupling. The SPP field induced by the SQD can be expressed as $E_{MNP}=[s_{\alpha }P_{MNP}]/[4\pi \varepsilon_{0}\varepsilon_{eff2}d^{3}]$ that is the mean value of the electric field operator ${\hat{E}}_{MNP}$, where $\varepsilon_{eff2}=[\varepsilon_{M}(\omega )+2\varepsilon_{0}]/[3\varepsilon_{0}]$. The operator can be split into two contributions ${\hat{E}}_{MNP}^{+}+{\hat{E}}_{MNP}^{-}$ evolving with positive and negative frequencies [29]. Based on the principle of second quantization for SPP field, we have $<\mu {\hat{E}}_{MNP}^{+}>=\underset{k}{\sum }g_{k}<a_{k}>$[26]. The above result is under the dipole approximation when the distance is large comparing to the radius of the MNP. However, if the distance is comparable to the radius of the MNP, we need to consider the multipole polarization in the MNP, including dipole, quadrupole, octopole, and so on. So the multipole polarization can be expressed as $P_{MNP,tot}=\sum_{n=1}\left(s_{n}\varepsilon_{0}\gamma_{n}R^{2n+1}P_{SQD}\right)/(\varepsilon_{eff1}d^{2n+1})$[30], where *s*_*n *_= (*n *+ 1)^2 ^for the polarization parallel to the axis of the complex system, *γ*_*n *_= [*ε*_*M*_(*ω*) *- ε*_0_]/[*ε*_*M*_(*ω*) + *ε*_0_(*n *+ 1)/*n*]. For simplicity we assume that the distance is larger than the radius of the MNP so that the dipole approximation (*n *= 1) is reasonable. In the dissipative system, the expectation value <*a*_*k *_> = *Tr*[*ρ a*_*k*_] of each SPP mode satisfies the equation, $\partial_{t}<a_{k}>=\left(\delta_{k}-i\gamma_{k}/2\right)<a_{k}>-g_{k}^{*}<\sigma_{-}>$. At steady state, we can obtain

(8) $$\sum_{k}\frac{{\left|g_{k}\right|}^{2}}{\delta_{k}-i\gamma_{k}/2}=\frac{\gamma {\left(\mu s_{\alpha }\right)}^{2}R^{3}}{4\pi \varepsilon_{0}\varepsilon_{eff1}\varepsilon_{eff2}d^{6}}.$$

Therefore, *η *= Re[*G*], *τ *= Im[*G*], where $G=[\gamma {\left(\mu s_{\alpha }\right)}^{2}R^{3}]/[4\pi \varepsilon_{0}\varepsilon_{eff1}\varepsilon_{eff2}d^{6}]$. We note that, here, *η, τ *~ *d*^-6^. So, it is reasonable that *g*_*k *_~ *d*^-3^. The verdict is in good agreement with the coupling strength between a two-level system and a single mode of SPP field [24,27]. In [9], Zhang et al. found that the interaction between an SQD and an MNP leads to the formation of a hybird exciton with the shifted exciton frequency and the decreased lifetime in which the SPP field is treated as a classical field rather than a quantized field. Here, we make a same conclusion under the quantized SPP field.

An experimental scheme to measure the two parameters is proposed by observation on the absorption spectrum of SQD in the system. Now, we consider an SQD in the vicinity of an Au MNP excited a weak signal light *E*_*s *_with frequency *ω*_*s*_. According to master equation *∂*_*t*_*ρ*_*SQD *_= -*i*[*H',ρ*_*SQD*_] + *ς'*_*SQD*_, where $H^{\prime }=\left(\omega_{ex}-\eta \right)\sigma_{z}-\mu \left(E_{s}\sigma_{+}e^{-i\omega_{s}t}+E_{s}^{*}\sigma_{-}e^{i\omega_{s}t}\right),\varsigma_{SQD}=\left(\gamma_{SQD}^{tot}/2\right)\times \left(2\sigma_{-}\rho \sigma_{+}-\rho \sigma_{+}\sigma_{-}-\sigma +\sigma_{-}\rho \right),\gamma_{SQD}^{tot}=\kappa +2\tau$, we have

(9) $$\partial_{t}p=\left[i\left(\eta -\omega_{ex}\right)-\gamma_{SQD}^{tot}\right]p-i\mu^{2}E_{s}e^{-i\omega_{s}t}w,$$

(10) $$\partial_{t}w=-2\gamma_{SQD}^{tot}\left(w+1\right)+2i\mu \left(E_{s}p^{*}e^{-i\omega_{s}t}-E_{s}^{*}pe^{i\omega_{s}t}\right),$$

where *p *= *μρ*_*ex*,0_, *w *= *ρ*_*ex*,*ex *_- *ρ*_0,0_.

The steady state solution can be obtained by setting the left-hand sides of Eqs. (9) and (10) equal to zero. Thus,

(11) $$w=-\frac{1+{\left(\omega_{s}+\eta -\omega_{ex}\right)}^{2}T^{2}}{1+{\left(\omega_{s}+\eta -\omega_{ex}\right)}^{2}T^{2}+8{\left|\mu E_{s}\right|}^{2}T^{2}},$$

(12) $$p=\frac{\mu E_{s}we^{-i\omega_{s}t}}{\left(\omega_{s}+\eta -\omega_{ex}\right)T+i},$$

where $T=1/\gamma_{SQD}^{tot}$. The polarization induced by the signal light can be expressed as $p=\varepsilon_{0}\chi E_{s}e^{-i\omega_{s}t}$[31], where *χ *is the total susceptibility to all order. We can obtain the total susceptibility: $\chi =\left({\left|\mu \right|}^{2}/\varepsilon_{0}\right)\times \left[T-\left(\omega_{s}+\eta -\omega_{ex}\right)T^{2}\right]/\left[1+{\left(\omega_{s}+\eta -\omega_{ex}\right)}^{2}T^{2}+8{\left|\mu E_{s}\right|}^{2}T^{2}\right]$. It can be expended in powers of the electric field *χ *= *χ*^(1) ^+ 3 *χ *^(3)^|*E*_*s*_|^2 ^+···, where

(13) $$\chi^{\left(1\right)}=\left({\left|\mu \right|}^{2}/\varepsilon_{0}\right)\times \frac{T-\left(\omega_{s}+\eta -\omega_{ex}\right)T^{2}}{1+{\left(\omega_{s}+\eta -\omega_{ex}\right)}^{2}T^{2}}$$

is the first-order (linear) susceptibility.

**Figure 2:**
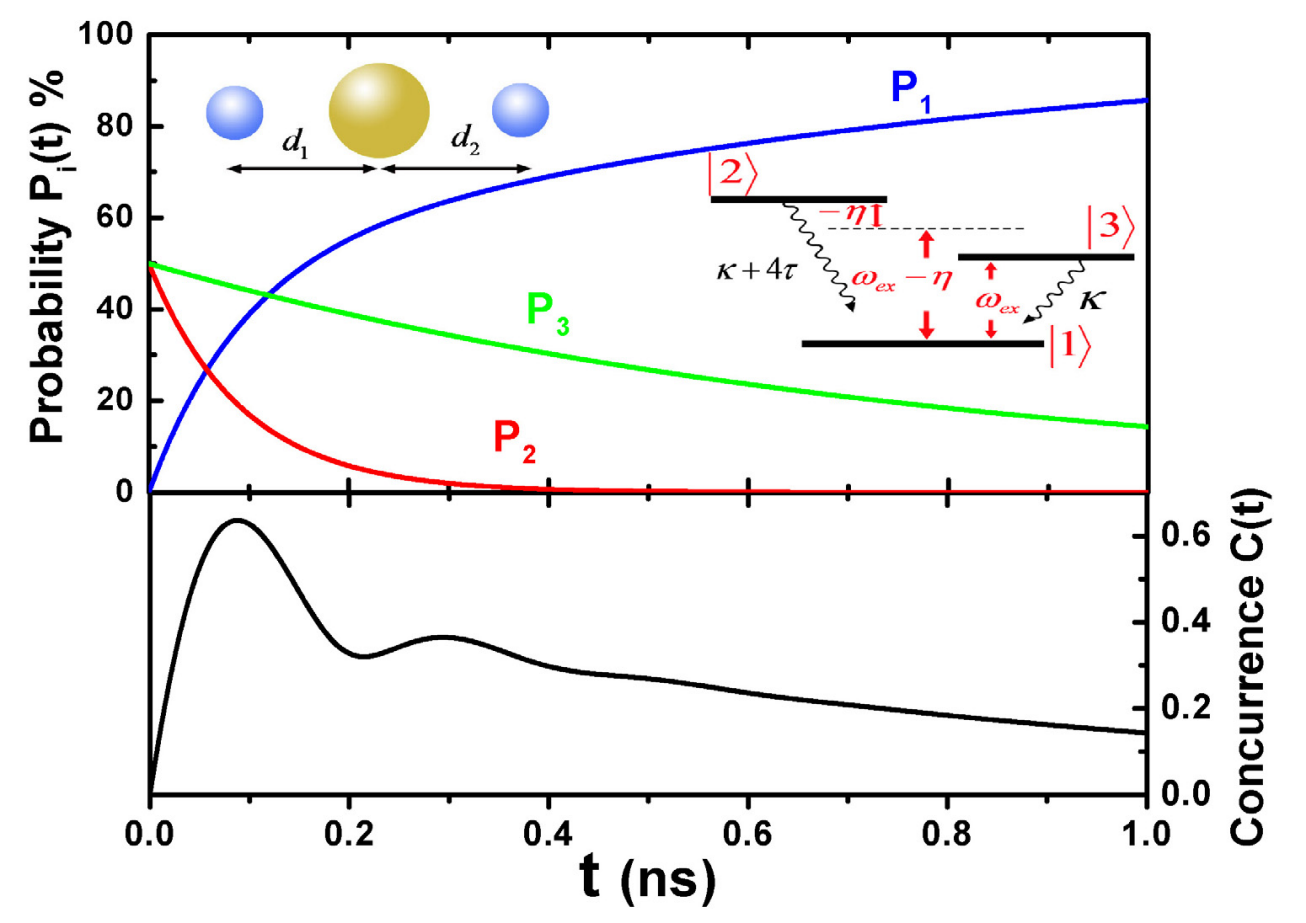
**The probability and the concurrence in one case**. The probability of each state, the concurrence of the two SQDs as a function of time when the initial state of the two SQDs is the state |*ex*, 0〉. The left inset shows a model composed of two SQDs and a MNP. The right inset shows the dissipation channels of the two SQDs.

In what follows, as an example, we consider a CdSe SQD with radius *r *= 3.75 nm [4] and an Au MNP with radius *R *= 7.5 nm. We use *ε*_0 _= 1.8, *ε*_*s *_= 7.2 [32] and the electric constant of Au $\varepsilon_{M}\left(\omega \right)=\varepsilon_{b}-\omega_{p}^{2}/\left[\omega \left(\omega +i\eta_{p}\right)\right]$ with ϵ_*b *_= 9.5, *ħ*_*ω *_= 2.8*eV, ħω*_*p *_= 9*eV, ħη*_*p *_= 0.07*eV *[22,33]. For the decay rate and dipole moment of the SQD, we take *κ *= 1.25 GHz and *μ *= *er*_0 _with *r*_0 _= 0.65 nm. Figure 2 shows the absorption spectrum of the SQD (the imaginary part of linear susceptibility Im[*χ*^(1)^]) as a function of the signal-SQD detuning for *d *= 30, 21, 18, 16 nm. We note that the absorption peak is shifting and broadening with the decreasing distance between the SQD and the MNP. The absorption peak shift represents the exciton energy shift, and the broadened peak implies the increased decay rate of SQD as a result of the presence of SPP field. So, the exciton energy shift *η *and the cross-decay rate 2*τ *can be obtained by observation of the absorption spectrum. As shown in Figure 2, the exciton energy shift (full width at half maximum) is about 6.5*κ *(2.5*κ*) for a small distance *d *= 16 nm.

## 4 Coupling of two SQDs

**Figure 3:**
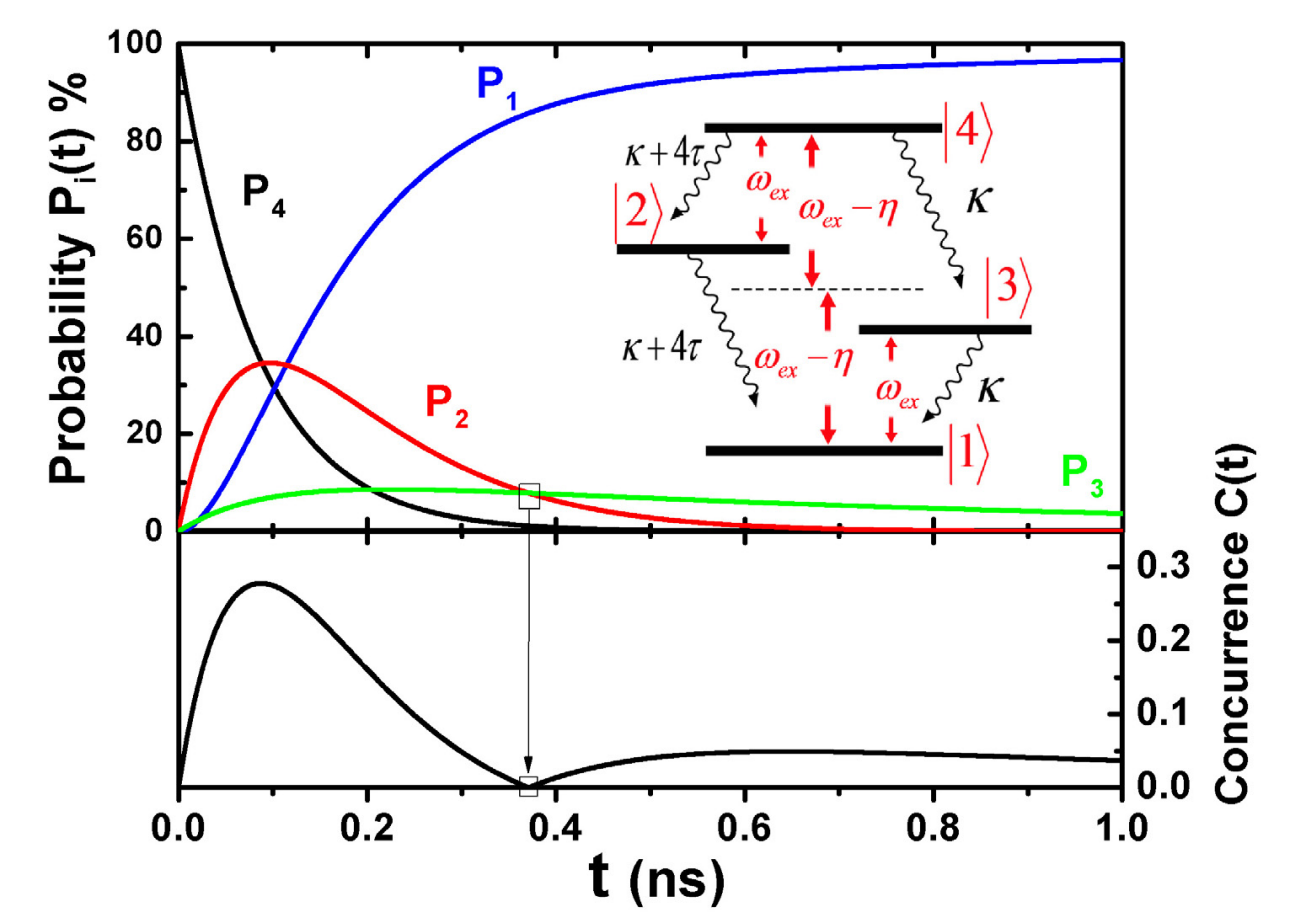
**The probability and the concurrence in another case**. The probability of each state, the concurrence of the two SQDs as a function of time when the initial state of every SQD is in their excited state. The inset shows the dissipation channels of the two SQDs.

We consider a simple model composed of two identical SQDs and an Au MNP for revealing the coupling between two SQDs induced by SPP field, as shown in left inset of Figure 2. The interaction between the two identical SQDs can be neglected safely in the absence of the MNP if the distance between them is very lager. When the distances between every SQD and the MNP are not equal (*d*_1 _≠ *d*_2_), we need to make a modification for the expression of two parameters *η, τ*. If one of the two distances changed, the expressions of the cross-decay rate and the coupling constant between the two SQDs need to be modified. As mentioned above, *g*_*k *_~ *d*^-3^. The expression of the cross-decay rate and the coupling strength can be rewritten as Im[*G*'] and Re[*G*'], respectively, where $G^{\prime }=[\gamma {\left(\mu s_{\alpha }\right)}^{2}R^{3}]/[4\pi \varepsilon_{0}\varepsilon_{eff1}\varepsilon_{eff2}d_{1}^{3}d_{2}^{3}]$. However, here, we assume that *d*_1 _= *d*_2 _= *d *for simplicity. In the SQDs' subsystem, we choose an adequate basic of SQDs' subsystem, i.e., $|1\rangle =|0,0\rangle ,|2\rangle =(1/\sqrt{2})\times (|ex,0\rangle +|0,ex\rangle ),|3\rangle =(1/\sqrt{2})\times (|ex,0\rangle -|0,ex\rangle ),|4\rangle =|ex,ex\rangle$. The four collective states are the eigenstates of the two coupling SQDs. The master equation of the SQDs' subsystem is given by

(14) $$\partial_{t}\rho =-i\left[H^{\prime \prime },\rho \right]+\varsigma_{SQD},$$

where *H*'' = -(*ω*_*ex *_- *η*) |1〉 〈1| - *η *|2〉 〈2| + *η *|3〉 〈3| + (*ω*_*ex *_- *η*) |4〉 〈4|, *ζ*_*SQD*_(*ρ*) = [(*κ *+ 4*τ*)/2] × [2(|2〉 〈4| + |1〉 〈2|)*ρ*(|4〉 〈2| + |2〉 〈1|)-(|2〉 〈2| + |4〉 〈4|)*ρ*-*ρ*(|2〉 〈2| + |4〉 〈4|)] + (*κ*/2) × [2(|1〉 〈3| -|3〉 〈4|)*ρ*(|3〉 〈1|-|4〉 〈3|)-(|3〉 〈3| + |4〉 〈4|)*ρ *- *ρ*(|3〉 〈3| + |4〉 〈4|)]. It shows two dissipated channels. The first term describes dissipation through one cascade channel |4〉 → |2〉 → |1〉 with fast decay rate *κ *+ 4*τ*. The second term describes dissipation through another cascade channel |4〉 → |3〉 → |1〉 with slow decay rate *κ *(see inset of Figure 3).

In order to illustrate the coupling of the two SQDs, we analyze the following two parameters: (1) The probability of the two SQDs being in the state |*i*〉, *P*_*i *_(*t*) = *ρ*_*i*,*i*_(*t*), for *i *= 1, 2, 3, 4. (2) The concurrence for quantifying entanglement of the two SQDs, $C\left(t\right)=\sqrt{{\left[\rho_{2,2}\left(t\right)-\rho_{3,3}\left(t\right)\right]}^{2}+4\text{Im}\;{\left[\rho_{2,3}\left(t\right)\right]}^{2}}$[17,34]. Here we use the parameters of the above section, and take *d *= 16 nm.

If the initial state of the two SQDs is prepared in a product state |*ex*, 0〉, only two dissipation channels |2〉 → |1〉 and |3〉 → |1〉 should been considered (see right inset of Figure 2). To obtain the probability of each state, Eq. (14) can be rewritten as $\partial_{t}\rho_{i,j}\left(t\right)=-i\underset{k}{\sum }\left({H^{\prime \prime }}_{i,k}\rho_{k,j}-{H^{\prime \prime }}_{k,j}\rho_{i,k}\right)+<i\left|\varsigma_{SQD}\right|j>$. According to the initial state density matrix *ρ*(0) = (|2〉 + |3〉)(〈2| + 〈3|)/2, we can obtain the the probability of each state and the concurrence. As shown in Figure 2, with the decrease of *P*_2_(*t*) and *P*_3_(*t*), the probability of the two SQDs in the state |1〉 increases. At about *t *= 0.08 ns, the concurrence ofthe two SQDs reaches the maximal value. In the figure of the concurrence, a weak oscillation is presented as a result of the coupling of the two SQDs.

**Figure 4:**
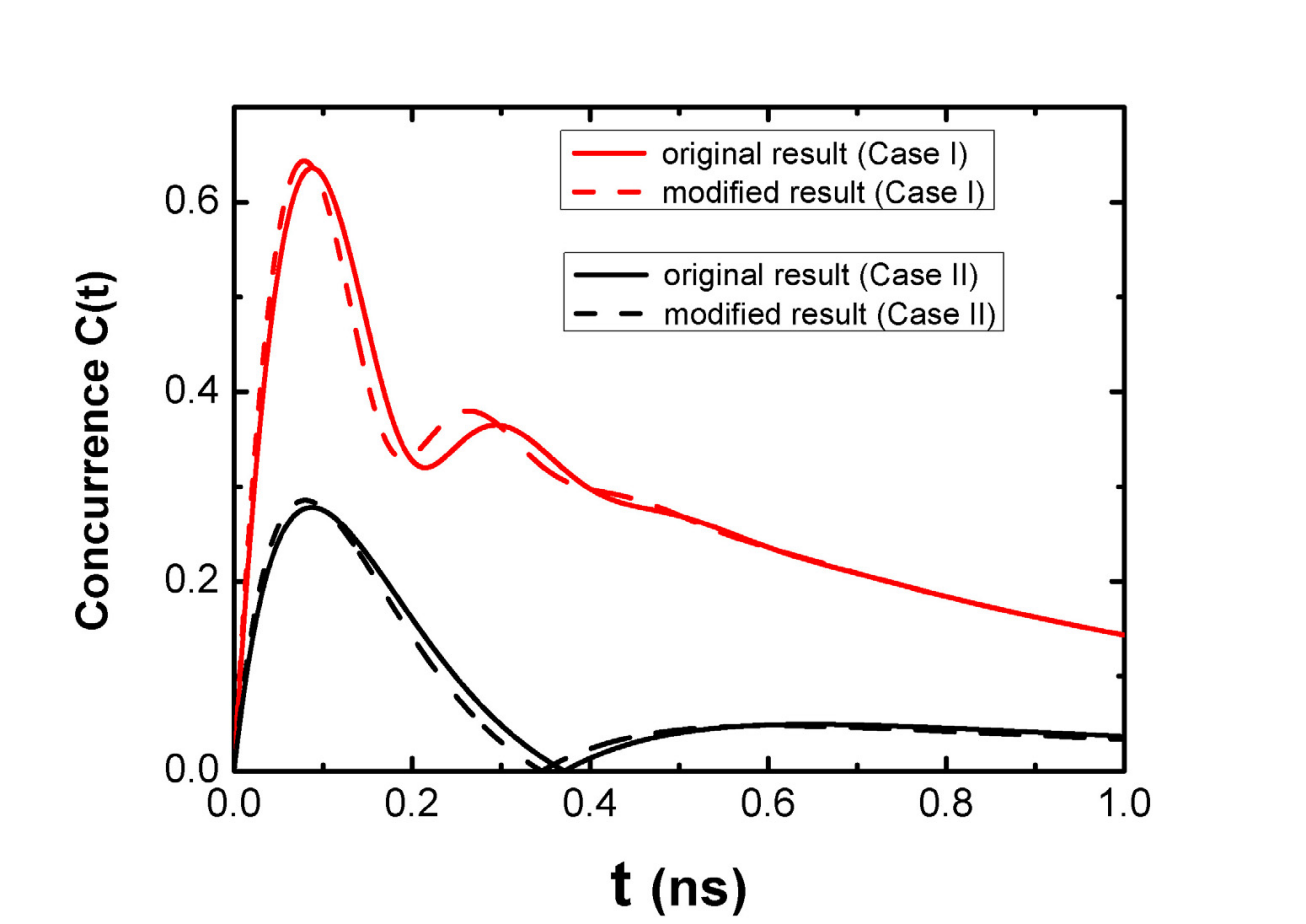
**Comparison between the original results and the modified results**. The concurrence as a function of time: the original results with a fixed distance *d *= 16 nm (solid curves), the modified results to reveal the dispersion effects of size distribution of the SQD (dash curves).

Another case is that the initial state is in another product state |*ex, ex*〉 (*ρ*(0) = |4〉 〈4|). Figure 3 shows the probability of each state, the concurrence as a function of time. It shows that the two SQDs can be entangled. Only at about *t*_0 _= 0.275 ns the concurrence is equal to zero (see the figure of the concurrence); and *P*_2_(*t*_0_) = *P*_3_(*t*_0_) (see the figure of probability). This is because two entangled states |2〉 and |3〉 make a product state |*ex*, 0〉 or |0, *ex*〉. The absence of the oscillation in the figure of the concurrence implies that the coupling of the two SQDs cannot play a role in the creation of the concurrence. In the two cases, we can generate the entangled state of the two SQDs because the quantized SPP modes are act as the platform of the energy transfer between the two SQDs. If the MNP is absent (*d *→ ∞ ), the coupling strength *η *and the cross-decay rate *τ *of the two SQDs are equal to zero so that the SQDs cannot be entangled. We can tune the concurrence of the two SQDs by changing the distance *d*. In our theoretical calculations presented above, we do not consider size distribution of the SQD. A numerical averaging of the obtained results for different spatial dispersions of the distance will give a perfect prediction of the dispersion effects on the concurrence. Because of size inhomogeneities of CdSe SQD, we assume that the position distribution density satisfies the Gaussian distribution $\rho \left(r\right)=exp\left[{-r}^{2}/\left(2\sigma^{2}\right)\right]/\left(\sqrt{2\pi \sigma }\right)$, with the the half-width of Gaussian distribution *σ *= 16*Å*. Figure 4 shows a comparison between the original results and the modified results considering the dispersion effects on the concurrence under the two cases. We can see the difference between the two results. The difference becomes slighter with decreasing *σ*. When the half-width *σ *is much smaller than the radius of the SQD, there is good agreement between the two results. Moreover, a stationary state with a high concurrence can be achieved by continuous pumping [17].

In conclusion, we have clearly demonstrated the interaction of SQDs and SPP field in MNP via a novel quantum transformation. The SPP field can induce the exciton energy shift and the decay rate modification of each SQD. The expressions of them is given by analysis. They can be measured by the designed scheme. Moreover, the coupling of two SQDs mediated by SPP field has been revealed strongly under two cases. With respect to the coupling among three or more SQDs, it is very significant for multipartite entanglement. The entanglement due to the light-matter interaction in the coupled SQD-MNP system may be applied in all-optical plasmon-enhanced nanoscale devices.

## Competing interests

The authors declare that they have no competing interests.

## Authors' contributions

YH finished the main work of this paper, including deducing the formulas, plotting the figures, and drafting the manuscript. KDZ participated in the discussion and provided some useful suggestion. All authors are involved in revising the manuscript and approved the final version.
